# A different background of arrhythmia in siblings with a positive family history of sudden death at young age

**DOI:** 10.1111/anec.12707

**Published:** 2019-10-14

**Authors:** Małgorzata Stępień‐Wojno, Maria Franaszczyk, Robert Bodalski, Mateusz Śpiewak, Rafał S. Baranowski, Jacek Grzybowski, Rafał Płoski, Zofia T. Bilińska

**Affiliations:** ^1^ Unit for Screening Studies in Inherited Cardiovascular Diseases The Cardinal Stefan Wyszynski Institute of Cardiology Warsaw Poland; ^2^ Department of Medical Biology The Cardinal Stefan Wyszynski Institute of Cardiology Warsaw Poland; ^3^ Department of Arrhythmia The Cardinal Stefan Wyszynski Institute of Cardiology Warsaw Poland; ^4^ Department of Radiology The Cardinal Stefan Wyszynski Institute of Cardiology Warsaw Poland; ^5^ Department of Cardiomyopathy The Cardinal Stefan Wyszynski Institute of Cardiology Warsaw Poland; ^6^ Department of Medical Genetics Warsaw Medical University Warsaw Poland

**Keywords:** basic, clinical, electrophysiology ‐ cardiac arrest/sudden death, molecular biology/genetics

## Abstract

We present two symptomatic sisters who had a positive family history of sudden death. None of them had structural heart disease. In the 25‐year‐old proband, complex ventricular arrhythmia, cardiac conduction system disease, and skeletal muscle weakness were found. Genetic examination showed a pathogenic intronic variant in the desmin gene in the proband only. In the elder sister with palpitations, complex ventricular arrhythmia (>46 000 ectopic beats) was removed by radiofrequency ablation. This family case shows that complex ventricular arrhythmia may have different background within one family, genetic examinations should be performed in a person with broadest spectrum of symptoms.

## 1 INTRODUCTION

A history of sudden cardiac death (SCD) of young individuals raises an important question among their offspring in the family, especially when the offspring starts being symptomatic and are uneasy about the extension of SCD risk to them. Desmin is an integral component of Z‐disk located within the sarcomere, both in heart and skeletal muscle, linking the Z‐disks to one another and neighboring sarcomeres, thus forming myofibrils, the basic unit of muscle fibers. Its role in pathogenesis of all types of cardiomyopathies is well established (Arbustini et al., 2006). Furthermore, *DES* gene mutations have been identified in sudden cardiac arrest survivors (Brodehl et al., 2013; Elliott et al., 2008; Stepien‐Wojno et al., 2018).

### 1.1 Family case description

**Figure 1:**
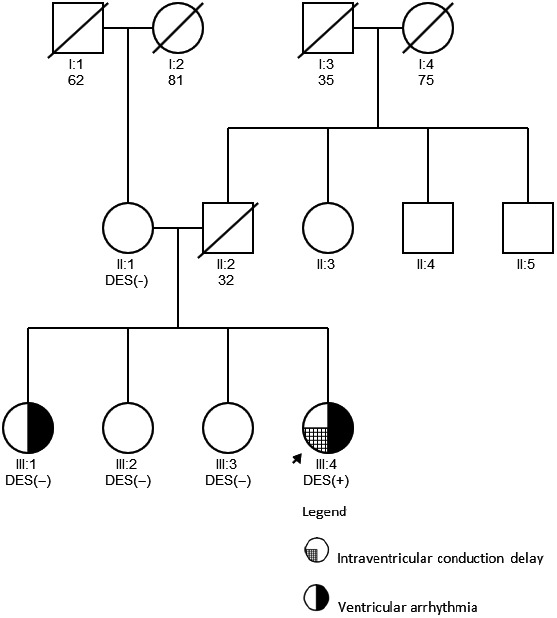
The pedigree of the family with *DES* c.735 + 3A>G mutation

We present a family with two symptomatic sisters, whose father died suddenly at the age of 32 years, and their paternal father died at the age of 35 years (Figure 1).

**Figure 2:**
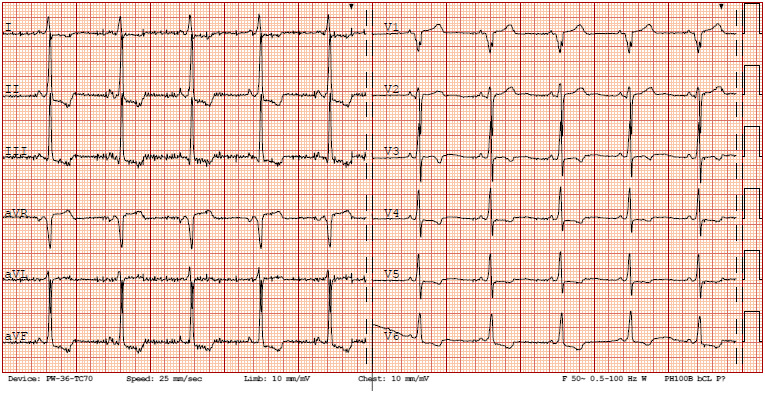
12‐lead standard electrocardiogram in the proband. Sinus rhythm 63/min, PR 130ms, nonspecific intraventricular conduction delay, QRS 119 ms, QTc 401 ms

**Figure 3:**
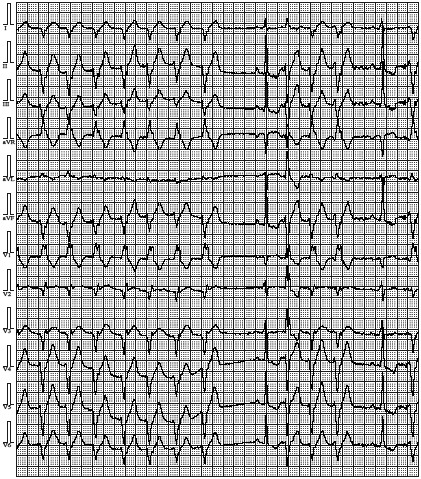
A strip of 12‐lead Holter ECG monitoring revealing nonsustained ventricular tachycardia of RBBB morphology in the proband

**Figure 4:**
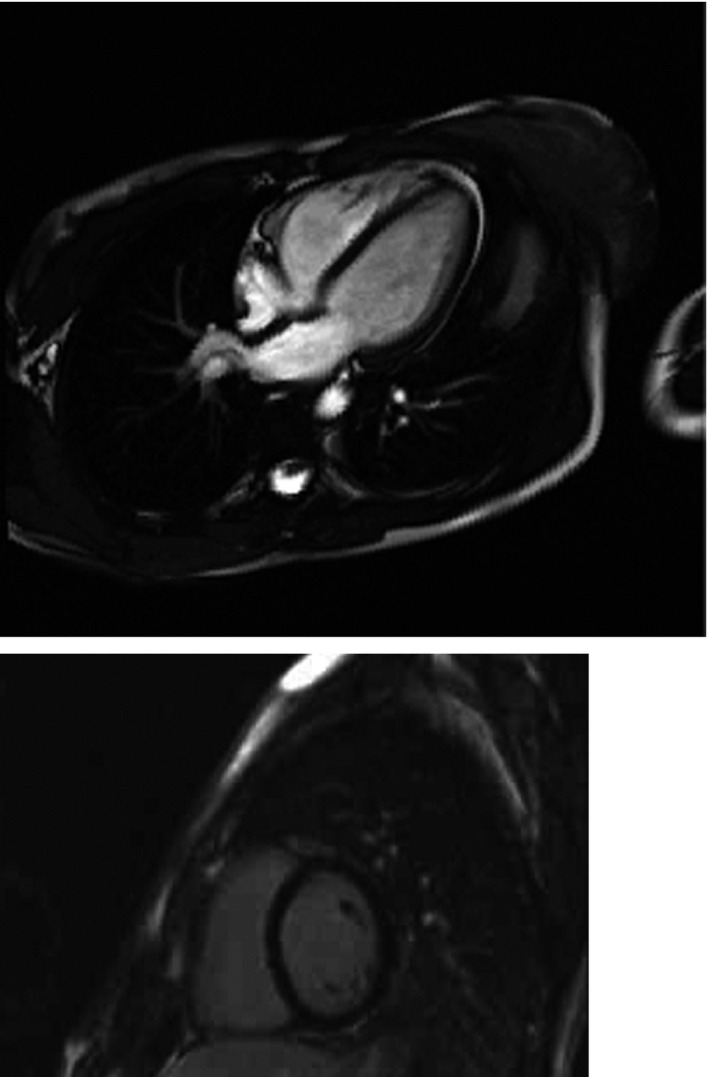
Cardiac magnetic resonance, 4‐chamber view revealing normal heart morphology

One of these sisters was 25 years old. Her medical history revealed episodes of postural hypotension and presyncope on effort and during palpitations. Moreover, she reported limbs’ weakness. Physical examination did not show any heart failure signs. A standard 12‐lead electrocardiogram showed sinus rhythm with heart rate 63 beats per minute and intraventricular conduction delay, QRS 119 ms (Figure 2). During 24‐hr ECG Holter, we recorded sinus rhythm 38–129 per min, average 58 per min, 5 pauses 2.1 s, infrequent, however complex ventricular arrhythmia: 4 nsVT and 289 single ventricular ectopic beats (Figure 3). Supraventricular arrhythmia was frequent, single, with 6,307 ectopic beats. Imaging examinations excluded structural heart disease and arrhythmogenic right ventricular cardiomyopathy (Figure 4.). Laboratory tests showed normal serum CK level, elevated both troponin T level 27.2 mg/ml and NT‐proBNP 413 pg/ml.

**Figure 5:**
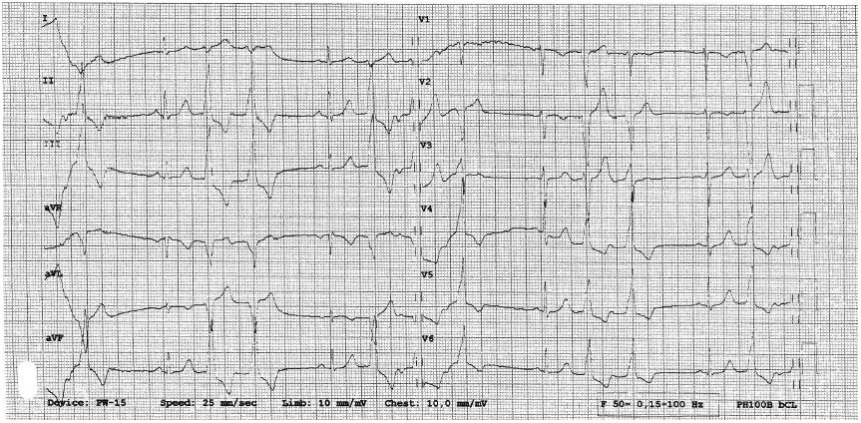
A standard 12‐lead ECG in the elder sister revealing sinus rhythm with frequent ventricular ectopy, also paired beats of LBBB morphology

At the same time, her elder sister, 31 years old, presented with symptoms of general weakness, palpitations, and dizziness. She did not have signs of heart failure, a standard 12‐lead ECG showed sinus rhythm with ventricular bigeminy, also paired ectopic beats (Figure 5). Echocardiogram and cardiac magnetic resonance did not show any abnormalities. 12‐lead 24‐hr ECG Holter monitoring revealed sinus rhythm mean 76 beats per min, and a lot of ventricular arrhythmia 46,799 beats/24 hr, including 16 episodes of nonsustained ventricular tachycardia (max. 5 beats, max. 105/min) with 2 morphologies of QRS, 16,969 paired ventricular beats. Her laboratory tests showed normal serum CK level of 48 U/l (26‐192U/l), normal both serum hs troponin T (<3 ng/L) and Nt‐proBNP of 86.2pg/ml (0‐125 pg/ml). Following the examination, the patient underwent radiofrequency (RF) ablation—applications in the right and left ventricular outflow tract.

We examined cardiologically other living first‐degree family members and did not find any abnormalities (Figure 1).

**Figure 6:**
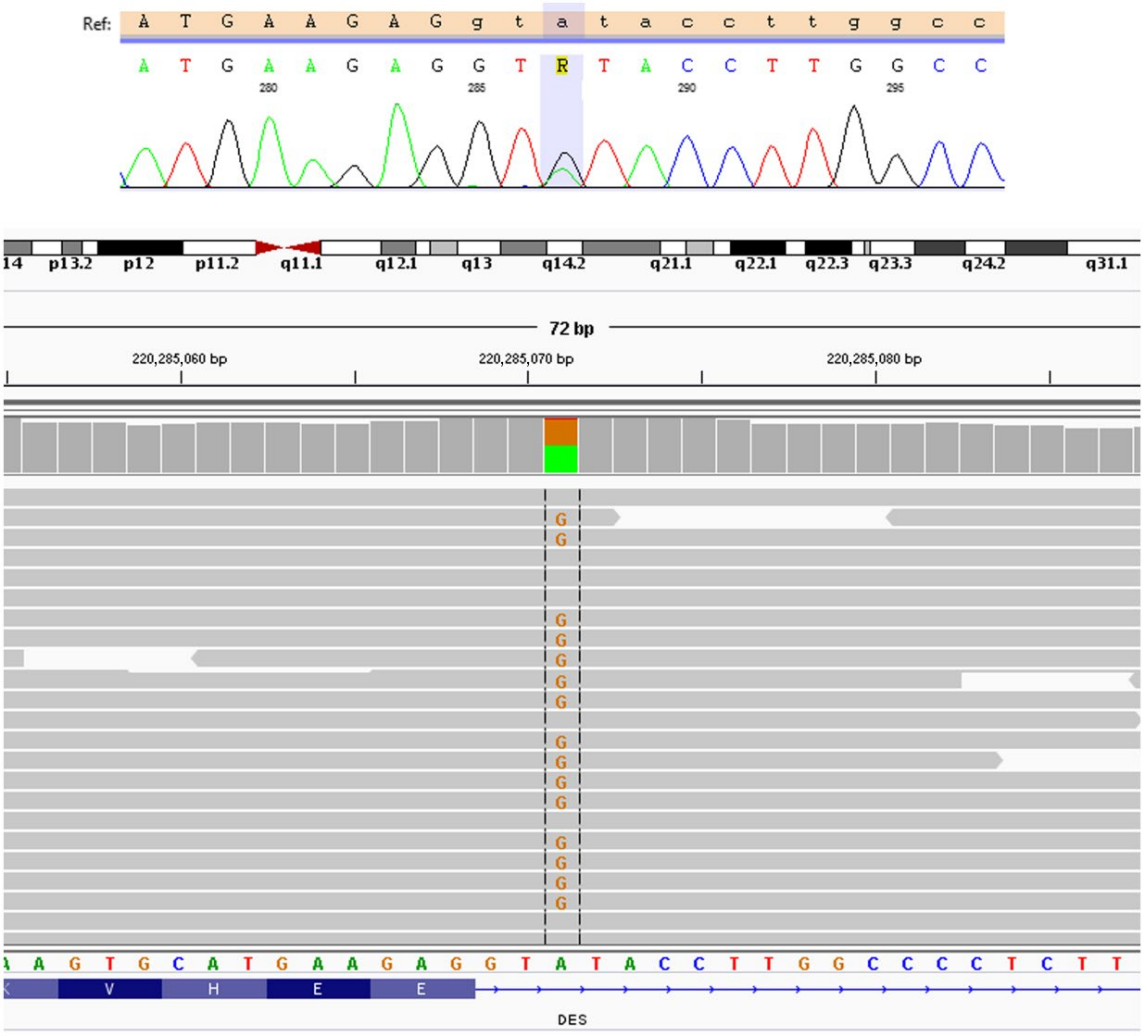
Chromatogram from direct sequencing by the Sanger method showing DES: NM_001927.3: c.735 + 3A>G variant found by TruSight One panel in the proband and in none of the examined relatives. IGV view of the mutation below chromatogram

It was decided to perform a genetic testing in younger sister due to presented symptoms and signs indicating involvement of the striated muscles and thus a syndromic disorder. DNA was extracted from the peripheral blood by phenol extraction. Next‐generation sequencing (NGS) in proband was performed using TruSight One (TSO) sequencing panel. Selected genetic variants identified with NGS were followed up in proband and relatives with Sanger sequencing. In proband, but not in her symptomatic sister, we identified the substitution of guanine for adenine in the third nucleotide of the splice donor site in intron 3 (IVS3 + 3A>G, the c.735 + 3A>G) in the desmin gene, classified as a pathogenic one by ClinVar. The variant was reported previously in the literature in association with desmin‐related myopathy, cardiomyopathy, and limb‐girdle muscular dystrophy (Dalakas et al., 2000; McDonald, Stajich, Blach, Ashley‐Koch, & Hauser, 2012; Park et al., 2000; Wahbi et al., 2012) (Figure 6). It is located in the 5’splice region of the *DES* and absent from large population databases (1000Genomes, ESP, and EXaC). Park et al showed in vitro that the variant may cause skipping of exon 3, what leads to an in‐frame deletion of 32 amino acids from the 1B segment of the alpha‐helical rod of desmin. Muscle biopsy in the 48‐year‐old variant carrier showed abundant intracytoplasmic inclusions in perinuclear and subsarcolemmal areas of some myofibers, that were strongly immunoreactive for desmin (Park et al., 2000). Deleterious effects were seen both in the cardiac and skeletal muscle of the carriers. Of note, three other variants impacting the same splice donor site c.735 + 1G>A, c.735 + 1G>T, c.735 + 1G>C (Goldfarb, Vicart, Goebel, & Dalakas, 2004; Gudkova et al., 2013; Park et al., 2000) have been reported and classified as likely pathogenic/pathogenic in relation to myofibrillar myopathy 1 or muscular dystrophy.

Although the temporary symptomatic status of the proband's sister made us to cautiously interpret the results of genetic testing in the family, the complete recovery of the proband's sister after RF ablation, maintained over 5‐year follow‐up confirmed a lack of causal relationship between the two types of ventricular arrhythmia present in sisters. In the meantime, in the proband we have been observing progressive course of the disease, especially with regard to cardiac conduction system disease and skeletal muscle weakness. During 5 years of follow‐up, broadening of the QRS complex, second‐degree atrioventricular block of Mobitz 2, episodes of ventricular arrhythmia were observed in our proband. Implantable cardioverter–defibrillator (ICD‐DR) was implanted. During the same period of time, there was no recurrence of arrhythmia, or appearance of conduction disturbances or muscle symptoms in the elder proband's sister after the ablation of arrhythmia.

This siblings’ case shows clinical difficulties in the diagnosis of the complex ventricular arrhythmia in the family with a history of sudden death and shows that genetic study should be performed in subjects with broadest clinical presentation.

## CONFLICT OF INTEREST

None declared.
